# Identification of Small Molecule Activators of BMP Signaling

**DOI:** 10.1371/journal.pone.0059045

**Published:** 2013-03-19

**Authors:** Karen Vrijens, Wenwei Lin, Jimmy Cui, Dana Farmer, Jonathan Low, Elodie Pronier, Fu-Yue Zeng, Anang A. Shelat, Kiplin Guy, Michael R. Taylor, Taosheng Chen, Martine F. Roussel

**Affiliations:** 1 Departments of Tumor Cell Biology, Memphis, Tennessee, United States of America; 2 Chemical Biology and Therapeutics, St. Jude Children's Research Hospital, Memphis, Tennessee, United States of America; 3 Institut National de la Santé et de la Recherche Medicale, U1009, Institut Gustave Roussy, Villejuif, France; University of Pittsburgh School of Medicine, United States of America

## Abstract

Bone Morphogenetic Proteins (BMPs) are morphogens that play a major role in regulating development and homeostasis. Although BMPs are used for the treatment of bone and kidney disorders, their clinical use is limited due to the supra-physiological doses required for therapeutic efficacy causing severe side effects. Because recombinant BMPs are expensive to produce, small molecule activators of BMP signaling would be a cost-effective alternative with the added benefit of being potentially more easily deliverable. Here, we report our efforts to identify small molecule activators of BMP signaling. We have developed a cell-based assay to monitor BMP signaling by stably transfecting a BMP-responsive human cervical carcinoma cell line (C33A) with a reporter construct in which the expression of luciferase is driven by a multimerized BMP-responsive element from the Id1 promoter. A BMP-responsive clone C33A-2D2 was used to screen a bioactive library containing ∼5,600 small molecules. We identified four small molecules of the family of flavonoids all of which induced luciferase activity in a dose-dependent manner and ventralized zebrafish embryos. Two of the identified compounds induced Smad1, 5 phosphorylation (P-Smad), Id1 and Id2 expression in a dose-dependent manner demonstrating that our assays identified small molecule activators of BMP signaling.

## Introduction

**Figure 1:**
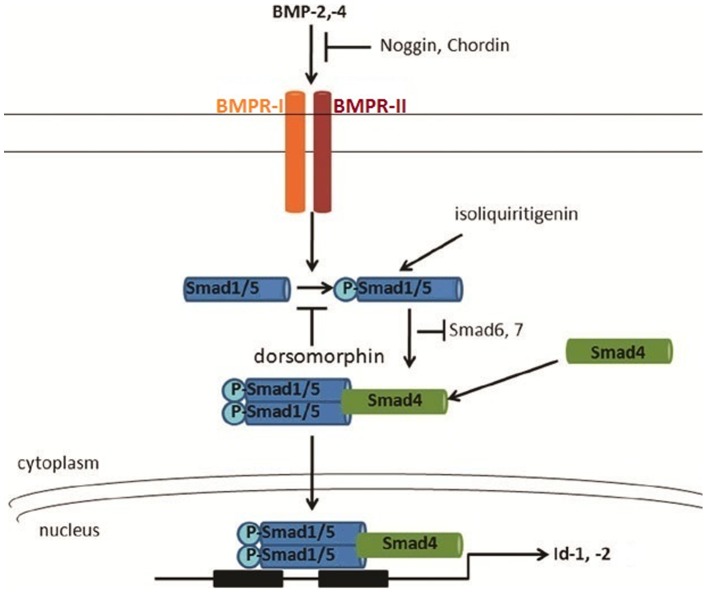
Canonical BMP signaling pathway. BMP canonical signaling is transduced by two type I (BMPR-IA and BMPR-IB) and two type II transmembrane serine/threonine kinase receptors, BMPR-II. BMPs bind to the heteromeric complex of type I and type II receptors. Subsequently, the type II receptor phosphorylates the type I receptor, which in turn facilitates phosphorylation of Smad1, 5 (R-Smads). R-Smads directly interact with the activated type I receptor and are released upon phosphorylation. Following release from the receptor complex, R-Smads complex with co-Smad 4 and translocate into the nucleus to modulate the transcription of target genes, including Id1 and Id2. Several natural extracellular proteins including Noggin and Chordin and the inhibitory Smads, Smad6 and Smad7, antagonize the BMP pathway. The small molecule dorsomorphin inhibits phosphorylation of Smads1, 5. Figure adapted from [15].

Bone Morphogenetic Proteins (BMPs) are a class of morphogens belonging to the transforming growth factor β (TGF-β) super-family, that were originally discovered because of their bone inducing capabilities [1], [2], [3]. The BMP family contains over 20 members with a wide array of functions, including embryonic patterning and development [4], stem cell renewal and differentiation [5], and tissue homeostasis [6]. BMPs signal through type I and type II serine/threonine kinase BMP receptors (BMPR) (Figure 1). Humans have three type I BMP receptors, BMPR-IA (Alk3), BMPR-IB (Alk6) and the type IA Activin (Alk2) receptor that binds activins and TGFβ as well as BMPs [7], [8]. BMPR-II is the only type II receptor [9]. Ligand binding induces the formation of a hetero-tetrameric receptor complex composed of two BMPR-IA and/or BMPR-IB receptors and two BMPR-II receptors and activates their kinase activity [10]. In turn, the activated complex recruits Smad1, 5, or 8 from the repressor Smad6 and phosphorylates (P) them at the C-terminus, an obligatory step in the canonical BMP signaling pathway [11], [12]. P-Smad1, 5, or 8 forms a complex with Smad4 that is trans-located to the nucleus and binds to BMP responsive elements (BRE) on the promoters of specific target genes, including the inhibitors of differentiation/DNA-binding 1–4, (Id1-4) [13]. Several endogenous antagonists of the pathway have been identified, including Noggin, which prevents BMPs from binding to the trans-membrane receptors (Figure 1) [14]. BMP signaling can also be mediated by Smad-independent pathways that are dependent on MAPK, and can lead to apoptosis or activation of downstream target genes through p38, JNK or Erk [15].

Medulloblastoma, a tumor of the cerebellum, is the most common malignant pediatric brain cancer [16], [17]. It is molecularly characterized into four groups; one of which sustains mutations that constitutively activate the Sonic Hedgehog (SHH) signaling pathway [18], [19]. BMP-2 and -4 signaling antagonizes SHH-dependent proliferation by inducing the irreversible differentiation of cerebellar granule neuron progenitors (GNPs) [20] and of medulloblastoma cells [21]. BMPs cause rapid turnover of the basic-helix-loop-helix transcription factor Atonal (Atoh1, Math1 in mice) [21] which is required for cerebellum development [14] and tumor formation [22]. GNPs from mice lacking Math1 fail to proliferate and animals are born with a cerebellum lacking the external germinal layer [14]. Deletion of Math1 in a mouse model of SHH-group medulloblastoma in which the SHH receptor Patched (Ptch) is mutated completely inhibits tumor development [22]. In mouse and human SHH-group medulloblastoma, genes in the BMP signaling pathway are down-regulated [21]. The SHH antagonist cyclopamine acts additively with BMP-2, and -4 to suppress GNPs and SHH-group medulloblastoma suggesting that BMP agonists could be used in combination with SHH-antagonists, both potentially at lower levels than alone, to reduce the potential side effects of the individual targeted therapies. The SHH-antagonist HH-Antag induces bone defects when administered to young tumor prone mice [23] and a patient treated with the SHH-antagonist GDC-0449 became resistant to the drug due to mutations in the targeted receptor Smoothened (SMO) [24]. Because recombinant BMPs are expensive to produce and are unlikely to cross the blood-brain barrier, small molecule activators of BMP signaling could provide a cost-effective alternative that are therapeutically available. In addition, the systemic administration of supra-physiological levels of BMPs as therapeutic agents induces severe side effects including inflammation, edemas and heterotopic bone formation [25]. Thus, small molecule BMP signaling activators may be more efficacious when used at lower levels mitigating the possible undesirable side effects.

Although several inhibitors of the BMP signaling pathway have been identified, including the synthetic antagonist dorsomorphin [26], to date, no small molecules have been shown to activate canonical BMP signaling. We here report our efforts to identify small molecule activators of the BMP signaling pathway.

## Methods

### Ethics Statement

Zebrafish were housed in an accredited facility of the Association for Assessment of Laboratory Animal Care (AALAC) in accordance with the National Institutes of Health guidelines. The Institutional Animal Care and Use Committee (IACUC) permit number A3077-01 of SJCRH approved all procedures in this study.

All animal work conducted to obtain primary medulloblastoma cells was performed under established guidelines and supervision by the St. Jude Children's Research Hospital's Institutional Animal Care and Use Committee (IACUC), as required by the United States Animal Welfare Act and the National Institutes of Health's policy, to ensure proper care and use of laboratory animals for research under the protocol # 378.

### Cell Lines and Tissue Culture

The human cervical carcinoma cell line C33A (ATCC, Manassas, VA) was maintained in Eagle's minimum essential medium (EMEM) supplemented with 10% FBS, 2 mM glutamine, 500 units/ml penicillin and 500 µg/ml streptomycin and grown at 37°C and 8% CO_2_. The C33A-2D2 subclone and C33A-C control cells we generated were grown in the same medium in the presence of 200 µg/ml Hygromycin B (Invitrogen, Carlsbad, CA). For the cell-based assay, C33A-2D2 and C33A-C cells were re-suspended in EMEM without phenol red, supplemented with 0.25% FBS, 500 units/ml penicillin and 500 µg/ml streptomycin, and 2 mM glutamine. C2C12 (ATCC, Manassas, VA) cells were grown in DMEM supplemented with 10% FBS, 500 units/ml penicillin and 500 µg/ml streptomycin and 2 mM glutamine, at 37°C and 8% CO_2_. To avoid depletion of the myoblastic population, cells were not allowed to grow to confluence but were passaged at a density less than 70%. NIH3T3 (ATCC, Manassas, VA) and HeLa (ATCC, Manassas, VA) cells were maintained in DMEM supplemented with 10% FBS and cultured at 37°C and 8% CO_2_. DAOY cells (ATCC, Manassas, VA) were maintained in EMEM supplemented with 10% FBS and cultured at 37°C and 8% CO_2_. Primary medulloblastoma cells were purified by Percoll density gradient from spontaneous tumors arising in *Ptch1+/−, Cdkn2c−/−* mice, as described previously [27]. For use in the assays, 2×10^6^ tumor cells were plated in 6-well dishes, allowed to attach overnight, and treated with BMP-4 or DMSO added to the culture medium. After 24 hrs, cells were trypsinized for 5 minutes at 37°C and harvested using an equal amount of ovomucoid solution. Samples were centrifuged, cell pellets frozen in dry ice and processed for western blotting as described below.

### Compound libraries selection

The screening library consisted of 5,600 (approximately 3,200 unique) approved drugs and chemicals with known biological activity (“St. Jude bioactives”). The library was assembled from 3 commercial suppliers: Microsource, Prestwick, and Sigma. The Microsource compounds included (a) the Spectrum collection, which contains 2,000 biologically active and structurally diverse compounds, including known drugs, experimental bioactives, and pure natural products, (b) the US Drug Collection, which contains 1,040 drugs that have reached clinical trials in the US and have been assigned USAN or US Pharmacopeia status; and (c) the Killer collection, which contains a reference set of 160 synthetic and natural toxic substances (http://www.msdiscovery.com/index.html). The Prestwick compounds include 1,120 small molecules selected for high chemical and pharmacologic diversity. Ninety percent of the collection is composed of known marketed drugs, and the remainder includes bioactive alkaloids or related substances. Human bioavailability and human toxicity data are available for most compounds (http://www.prestwickchemical.fr/index.php?pa=26. Accessed 2013 February 13). The Sigma LOPAC^1280^ (Library of Pharmacologically Active Compounds) collection reflects the most commonly screened targets in the drug discovery community, including marketed drugs, failed development candidates, and "gold standards" that have well-characterized activities (http://www.sigmaaldrich.com/catalog/product/sigma/lo1280?lang=en&region=US. Accessed 2013 February 13) [28].

### High Throughput Screening (HTS)

C33A-2D2 cells were seeded into white, solid-bottom, tissue culture-treated, 384-well polystyrene plates at a density of 5×10^3^ cells per well in 25 µl media. Compounds, BMP-4 (positive control), or DMSO (negative control) were transferred with a V&P 384-well pintool at 30 nl/well to give a final compound concentration of 12 µM into individual wells. The final positive BMP-4 control concentration was 100 ng/ml and the final DMSO concentration was 0.12%. The assay plates were then incubated overnight at 37°C, 5% CO2, 95% relative humidity followed by luminescence assay for luciferase reporter activity with SteadyLite HTS reagent. Activity data were normalized to 100 ng/ml of BMP-4 as 100% activation and 0.12% DMSO as 0% activation. For unique hits identified in the primary screening, dose response (DR) assays were implemented in triplicate against the C33A-2D2 cells in the same HTS setting at ten different concentrations, following a 3 fold dilution scheme from 56 µM down to 2.8 nM. Similarly, a BMP-4 dilution series from 100 ng/ml to 0.2323 fg/ml was used as control. To measure luciferase activity, we used a luminescent SteadyLite assay, as per manufacturer's instructions (PerkinElmer, manual 44-73605, Waltham, MA). Cells were lysed after a 24 hr treatment period by addition of SteadyLite reagent and luminescence was measured using a 2102 EnVision plate reader (PerkinElmer, Waltham, MA).

### Immunoblotting

C33A-2D2 cells were seeded into clear 6-well plates at a density of 4×10^5^ cells per well in 2 mL of medium. Cells were serum-starved for 6 hours and treated or not with each of the four compounds at concentrations of 5, 10, 18 and 24 µM for 24 hours. 10 ng/ml human recombinant BMP-4 (R&D systems, Minneapolis, MN) and 0.1% DMSO were included as positive and negative control, respectively. After collection, cells were homogenized in RIPA lysis buffer (50 mM Tris, pH 7.4; 150 mM NaCl; 0.1% SDS; 0.5% Sodium deoxycholate and 1% Triton X100), to which a cocktail of protease inhibitors (1 mM PMSF; 38 µg/ml aprotinin, 10 mM β-glycerophosphate; 1 mM NaF and 100 µM NaVO_4_) was added immediately before use. Proteins were quantified using a bicinchoninic acid (BCA) protein assay reagent (Pierce, Rockford, IL) according to the manufacturer's instructions. 20 µg of protein per sample were analyzed using a 10% SDS-PAGE gel for the detection of P-Smad1, 5 and Smad1,5 and a 12% SDS-PAGE gel for Id1 and Id2 detection. After electrophoresis, proteins were transferred to PVDF membranes. Membranes from the 10% gels were blocked in 5% milk/TBS-Tween 2 hours at room temperature (RT), incubated with a rabbit monoclonal antibody anti-P-Smad1, 5 (Cell Signaling # 9516S, Danvers, MA) at 1/500 dilution in 5% BSA TBS/Tween overnight (O/N) at 4 °C or a rabbit polyclonal antibody to Smad1, 5 (SC#6031-R) or to Actin (SSC#1615) (all from Santa Cruz Biotechnology, Santa Cruz, CA). We generated a rabbit polyclonal antibody raised against P-Smad1, 5 by immunization of a C-terminal phospho-peptide linked to KLH [VLTQMGSPLNPISS(P)VS(P)], in which the two serines at positions 463 and 465 were phosphorylated. This antibody was used at 1/3000 dilution. For the detection of Id1 and Id2, membranes from the 12% gels were blocked in 5% BSA in TBS/Tween 20 for 2 hours at RT, and incubated O/N in 5% milk/TBS/Tween 20 containing rabbit polyclonal antibodies to Id1 (SC#488), Id2 (SC#489) or Actin (C-11) at 1∶500 dilution (all from Santa Cruz Biotechnology, Santa Cruz, CA). Smad-independent BMP signaling was analyzed by (P)-Erk1/2 activation, with primary antibodies against Erk1/2 (MK1, Santa Cruz Biotechnology, Santa Cruz, CA) and P-Erk1/2 (Thr202/Tyr204) (#9101, Cell Signaling, Danvers, MA) at 1∶1,000 dilution. Incubation with primary antibodies was followed by incubation with species-specific secondary antibodies for 2 hours (anti-rabbit IgG horseradish peroxidase-linked whole antibody dilution 1∶2500 (GE Healthcare, Waukesha, WI), or anti-goat IgG HRP antibody dilution 1∶2500 (Invitrogen/Life Technologies, Grand Island, NY) coupled with horseradish peroxidase and proteins were detected by enhanced chemiluminescence (Perkin-Elmer, Waltham, MA). P-Erk levels were quantified by scanning the films from three independent experiments, each normalized to Erk levels as control using Image J software.

### Alkaline phosphatase (ALP) assays

C2C12 cells were seeded into 24-well polystyrene plates at a density of 2×10^3^ cells per well in 500 µl of medium. Cells were allowed to adhere overnight, and were stimulated with increasing concentrations of compounds (1 µM to 10 µM). As positive control we used a dilution series of human recombinant BMP-4 (R&D Systems) with concentrations ranging from 50 ng/ml to 5 ng/ml. Cells were incubated for 6 days, after which we measured levels of ALP. Cells were washed twice with PBS, and 250 µl lysis buffer was added to each well. Plates were incubated for 10 minutes at 4°C under shaking conditions, 250 µl of luminescent substrate (Sensolyte, Anaspec, Fremont, California) was added, and plates were incubated an additional 30 minutes at 4°C in the dark. Luminescence was measured using a Synergy 2 plate reader (Biotek, Winooski, VT).

### Immunohistochemical assays

To examine ALP activity histochemically, 2×10^4^ C2C12 cells were plated in 6-well plates and allowed to grow overnight in medium with 10% FBS. The next morning, medium was replaced with fresh medium containing 5% FBS. After 3 hours, BMP-4 or compounds were added, and cells were cultured for 6 days. Cells were fixed for 10 minutes with 3.7% formaldehyde at room temperature, washed with PBS and incubated for 20 minutes with 0.1 mg/ml naphtol AS-BI alkaline solution, 0.1 mg/ml Fast Blue benzamide (FBB)-alkaline solution and 2 µM sodium nitrite solution (all reagents from Sigma-Aldrich, St. Louis, MO). After 20 minutes, cells were washed with PBS and images taken using a Canon Powershot A650 AS.

### Zebrafish ventralization assay and morpholino injections

Wild-type zebrafish (*Danio rerio*) embryos of the TL strain were produced by natural spawning and maintained in egg water (0.03% Instant Ocean) at 28.5 °C. Embryos at 2 hpf were placed in 24-well plates (n = 15 per treatment) and treated with compounds at a concentration range of 1 µM to 80 µM. At 24 and 50 hpf, embryos were manually dechorionated and imaged. Morpholino knockdown of chordin was performed as previously described [29]. Briefly, embryos at the 1 to 2-cell stage were microinjected with approximately 8 ng of chordin morpholino, manually dechorionated, and imaged at 24 hpf.

## Results and Discussion

Current treatment strategies for patients with medulloblastoma are often insufficient, since 30% of patients succumb to their disease and survivors suffer from severe neurocognitive side effects. BMP-2 and BMP-4 irreversibly induce the differentiation of SHH-subgroup medulloblastoma in mouse models [21]. However, they cannot be used in a clinical setting to treat pediatric brain tumors because in addition to their cost, the size of recombinant BMP proteins prevent their penetration across the blood brain barrier [30]. Dura fibrosis was observed in pediatric patients treated with BMP-2 [31]. This has prompted us to identify small molecule agonists/activators of the BMP signaling pathway that could be used to treat medulloblastomas of the SHH-subgroup ideally in combination with SHH antagonists. We developed a cell-based high throughput screening (HTS) assay and several secondary assays to identify compounds that activate BMP signaling. We here report a screen of a bioactive library of approximately 5,600 individual compounds. We identified four small molecules of the family of flavonoids including two flavones and two chalcones that induced luciferase activity in a dose-dependent manner and ventralized zebrafish embryos, although only the two flavones activated BMP signaling by inducing Smad1, 5 phosphorylation, as well as Id1 and Id2 protein expression in a dose-dependent manner.

### High Throughput Screen cell-based assay

**Figure 2:**
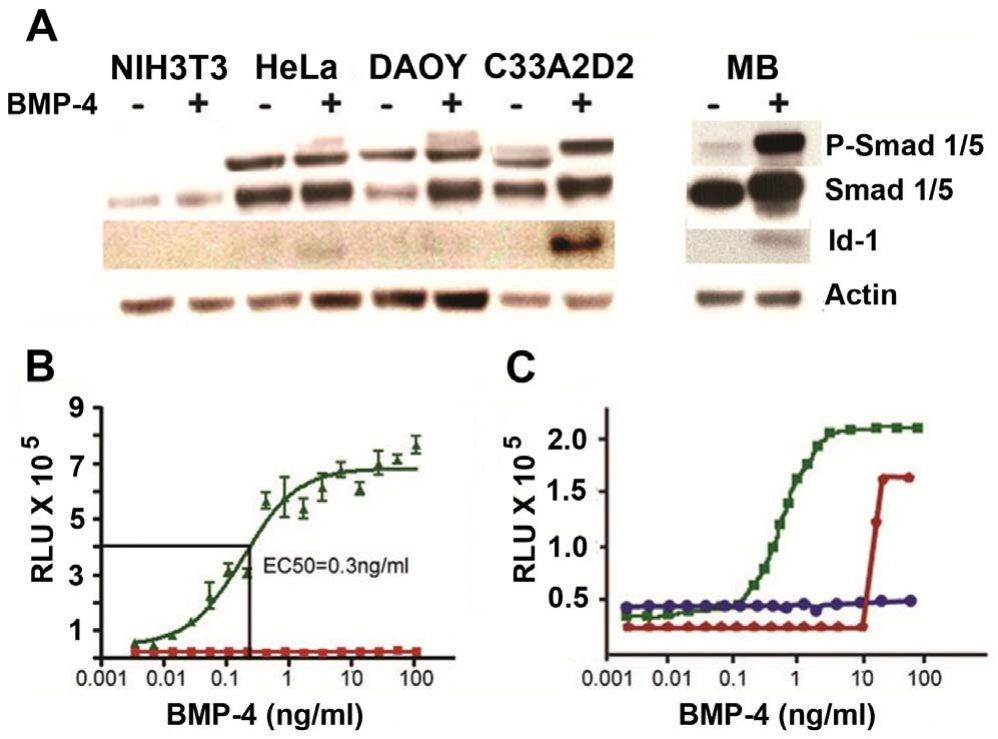
Development and validation of a cell-based assay. (**A.**) Response of different established cell lines NIH3T3, HeLa and DAOY to stimulation for 24 hrs with (+) or without (−) 20 ng/ml BMP-4. The clonal cell line C33A-2D2, showed induction of phosphorylation of Smad1, 5 and of Inhibitor of DNA binding 1 (Id1) expression in response to BMP-4 treatment. Primary medulloblastoma cells (MB) from *Ptch1^+/−^/Cdkn2c^−/−^* mice are shown as a positive control. (**B.**) Response of C33A-2D2 and C33A-C control cells to BMP-4 measured as raw luminescent activity (RLU). C33A-2D2 displays a saturated sigmoidal dose-response curve in response to BMP-4 stimulation with an EC_50_ value of 0.3 ng/ml BMP-4 (green curve). C33A-C (empty vector) is completely unresponsive to BMP-4 treatment (red curve). Both cell lines were treated with a dilution series (1/3) of BMP-4 from 100 ng/ml as top concentration. 24 hrs later, luciferase activity was assayed using Steady-Lite reagent. (**C.**) Antagonistic properties of the endogenous protein Noggin (red line) and the small molecule dorsomorphin (blue line) on BMP-4 (green line) in C33A-2D2 cells. Both molecules completely inhibited Id1 promoter activity at lower concentrations of BMP-4. At higher concentrations of BMP-4 (>10 ng/ml) Noggin could no longer compete with BMP-4 to prevent it from binding to the BMP receptors. The green line represents 1 to 3 dilution series of BMP-4 with the top concentration of 100 ng/ml. The red line represents BMP-4 dilution series containing a fixed concentration of Noggin at 250 ng/ml. The blue line represents a BMP-4 dilution series containing a fixed concentration of 10 µM dorsomorphin.

We developed a clonal reporter cell line in which luciferase activity was robustly induced by BMP-4 in a dose-dependent manner. The human cervical carcinoma clonal line, C33A-2D2 gave a robust BMP-4 response and low background compared to other cell lines including NIH3T3, HeLa, DAOY and primary SHH- medulloblastoma cells (Figure 2A). C33A cells were transfected with plasmids modified from a previously described reporter [32] that contained either a multimerized BMP-responsive element (BRE) linked to luciferase (pGL3-BRE-Luc) or an empty vector control (pGL3-Luc) [33]. After selection, hygromycin-resistant cells were subcloned by limiting dilution. The C33A-2D2 subclone induced luciferase levels in a BMP-4 dose-dependent manner (Figure 2B) with an EC_50_ value of 0.3 ng/ml. Although initially we evaluated the previously published C3H10T1/2 cell line [32], the EC_50_ for BMP-4 was higher than the current line at 8 ng/ml (Figure S1). In contrast, the control line C33A-C, stably expressing the control reporter construct, did not show induction of luciferase, as expected (Figure 2B). Addition of Noggin or dorsomorphin inhibited the luciferase activity induced by BMP-4, indicating that the assay specifically measured the activation of BMP signaling (Figure 2C).

We adapted the cell-based assay for HTS in 384-well plate format. The assay performed well over a 3-day validation period involving three independent triplicate experiments: average signal to noise was 10, average z-prime was 0.66 [34], and the maximum fold deviation from median EC_50_ for the positive control, BMP-4, was 1.18. The activity of compounds was calculated as a percent of activation using the following formula: 100* (compound–DMSO)/ (BMP-4–DMSO) in which measured values were log_10_-transformed luciferase Relative Luminescence Units (RLU).

### High Throughput Screen of a library of bioactive compounds

**Figure 3:**
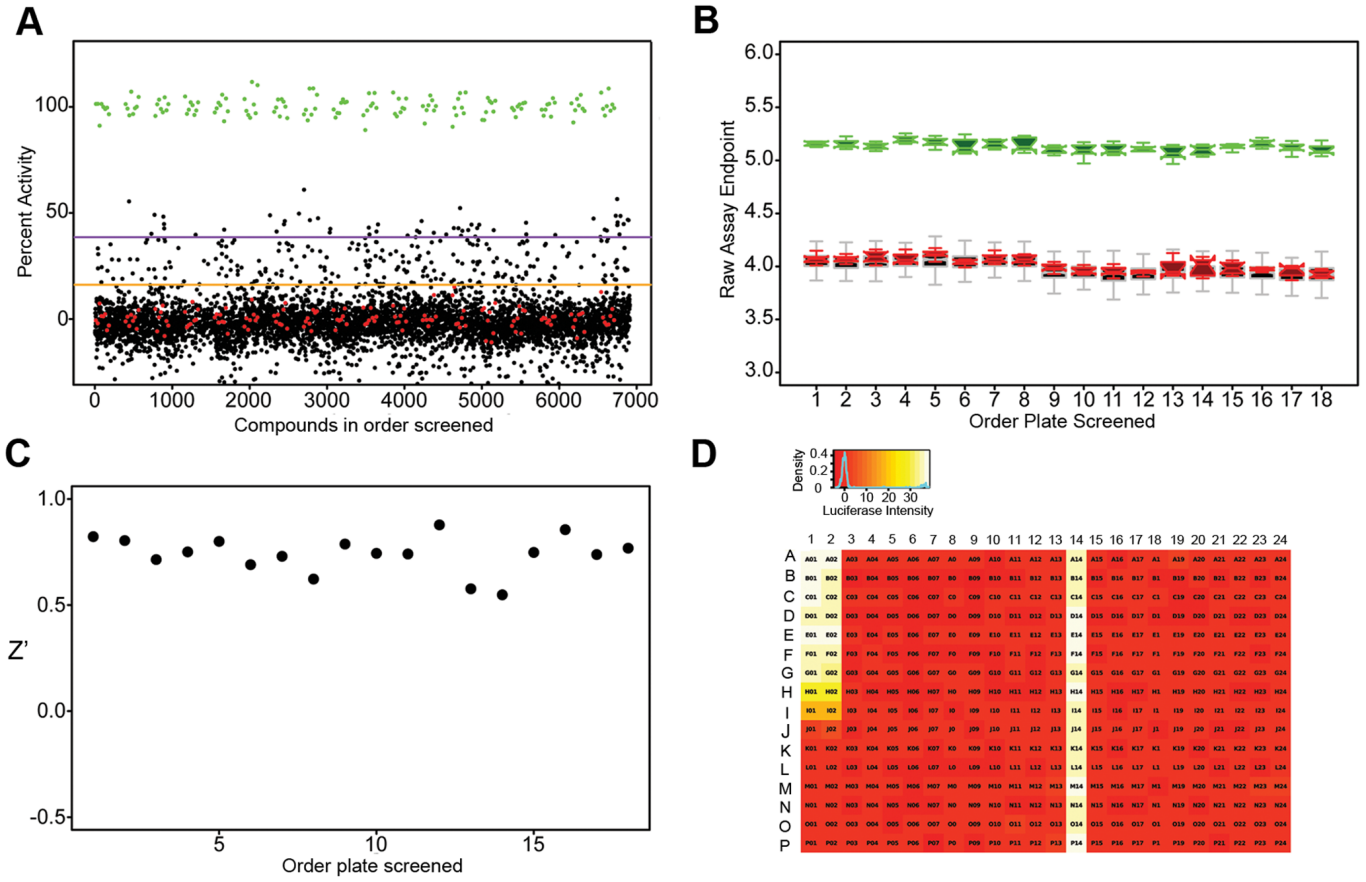
Cell-based assay quality control metrics. (**A.**) Percent activity scatterplot for all bioactive compounds screened at single point (12 µM) concentration in C33A-2D2 cells. green: positive controls (100 ng/ml of BMP-4); red: negative controls (DMSO); black: screening compounds. Orange line and purple lines represent the 95^th^ and 99^th^ activity percentiles, respectively, of the screening compound population. (**B.**) Boxplot of the positive control (green), negative control (red), and screening compound populations (black with gray outline). (**C.**) z-prime in the order of plates screened. (**D.**) Heatmap of well activity z-scores averaged over all plates in this screen. Columns 1 and 2 were reference dose-response curves for BMP-4 (100 ng/ml top concentration, followed by 1 to 3 dilutions down the column). Column 13 and 14 were the negative and positive controls, respectively. There were no significant plate artifacts in this assay.

We used the optimized assay to screen the ‘bioactives’ compound collection using a fixed concentration of 12 µM. BMP-4 (100 ng/mL) and DMSO (0.12%) on each plate as positive and negative controls, respectively. Data was analyzed using a custom informatics application (RISE). The assay demonstrated good discrimination between positive and negative controls, maintained stable signal, and lacked any significant plate artifacts (Figure 3A–D).

Four compounds had activity greater than 50%. One of these, the potent CDK inhibitor kenpaullone (SJ000287948), was excluded from further study. Thirty-two compounds (1% of the unique molecules screened), with activation of BMP signaling between 40–60% relative to BMP-4 (set at 100%), were selected for further study. These compounds were subjected to dose-response studies to determine their potency using the same assay as the primary screen and a top concentration of 56 µM. Selectivity was measured by counter-screening the same dilution plates against the C33A-C line, which measures non-specific luciferase activity. Of these 32 compounds, 19 had potencies below 10 µM with EC_50_ values ranging from 0.7 to 9 µM, whereas 7 compounds had EC_50_ values ranging from 12 to 56 µM and 6 compounds did not induce luciferase in a dose-dependent manner. Thus 60% of the initial active compounds were considered validated hits.

**Figure 4:**
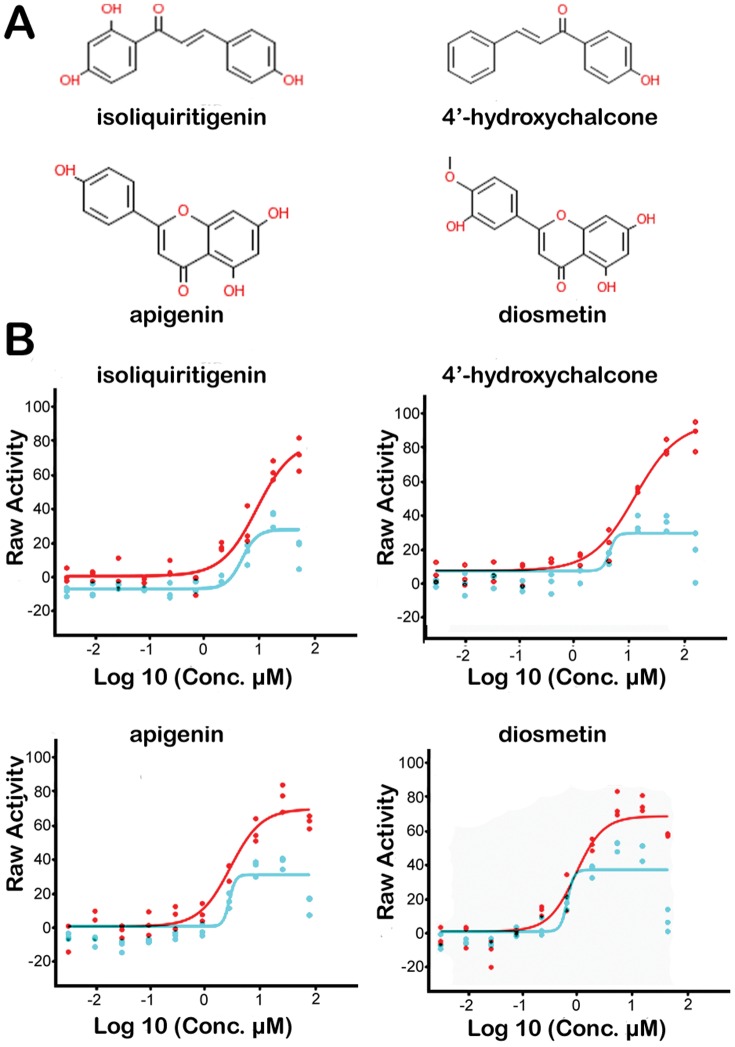
Structure and activity of identified hits. (**A.**) Chemical structures of the four compounds. (**B.**) Luciferase activity in C33A-2D2 cells (red curve) and C33A-C empty vector cells (blue curve) for 1/3 dilution series of compounds, with 56 µM as top concentration.

Among the 19 validated hits, 4 showed specific activation of BMP signaling, with a good differential in RLU between C33A-2D2 and C33A-C cells. All 4 validated hits were flavonoids, two were chalcones and two were flavones. Chalcones included SJ000286237, called isoliquiritigenin, that had a potency of 10 µM with 80% efficacy and SJ000286396 called 4′-hydroxychalcone with a potency of 10 µM with 90% efficacy (Figure 4, Table 1). The other two small molecules were flavones: SJ000287098, called apigenin, had a potency of 3 µM with 75% efficacy, whereas SJ000286673, called diosmetin, had a potency of 1.5 µM with 80% efficacy (Table 1). Chalcones are a group of aromatic enones that are natural dietary agents. Both chalcones and flavones were shown to have anti-tumor activity [35], [36]. However, these effects could possibly be attributed to the interaction with Cytochrome P450 (CYP) genes that metabolize the compounds into other products [37], or to inhibition of specific CYP genes [38]. Therefore, these compounds are not suitable for the further development of small molecules as potential therapeutic drugs.

**Table 1:** Effects of chalcones and flavones on BMP signaling.

compound class	compound name	SJ number	EC_50_ DR primary assay (µM)	WB C33A-2D2	zebrafish
chalcone	isoliquiritigenin	SJ000286237	8.647	+	ventralized
chalcone	4′-hydroxychalcone	SJ000286396	5.197	+	ventralized
flavone	diosmetin	SJ000286673	2.031	−	ventralized
flavone	apigenin	SJ000287098	0.952	−	ventralized
flavone	biochaninA	SJ000286709	1.485	−	normal
flavone	luteolin	SJ000287414	3.240	−	normal
flavone	biochaninA diacetate	SJ000287097	5.286	−	normal

(SJ) St Jude Children's Research Hospital; (DR) dose response; WB: Immunoblotting detecting activation of BMP signaling, phosphorylation of Smad1, 5, and expression of Id1 and Id2 in a dose-dependent manner; (+): significantly different from DMSO; (­): no significant difference from DMSO control (as calculated with student's t-test). Fold induction was calculated using Image J software. Phenotype of wild type zebrafish embryos was analyzed after treatment with compounds from 2 hpf until 30 hpf.

### The two chalcone compounds but not the two flavones activate BMP signaling

**Figure 5:**
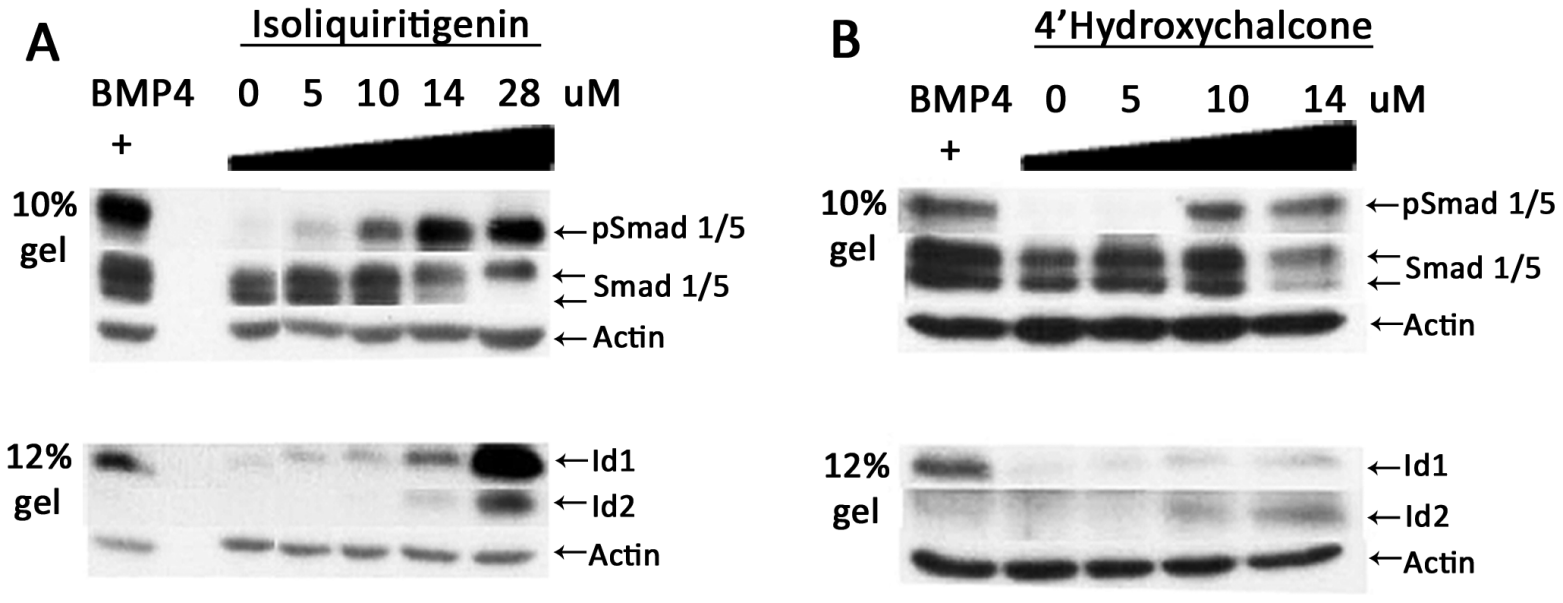
Activation of BMP signaling by the two chalcones isoliquititigenin and 4′-hydroxychalcone. C33A-2D2 cells were treated either with BMP-4 (+, 10 ng/ml) or increasing concentrations of either isoliquiritigenin (**A**) or 4′-hydroxychalcone (**B**). Proteins were separated on either 10% or 12% PAGE gels and immunoblotted with antibodies to phosphorylated Smad-1, 5, total Smad1, 5, 8, Id1 and Id2. Actin was used as loading control.

Following 24 hrs treatment, the two chalcones, isoliquiritigenin and 4′-hydroxychalcone activated Smad1, 5 phosphorylation, as well as Id1 and Id2 protein expression in a dose-dependent manner (Figure 5). The two other flavonoids, apigenin and diostemin that induced luciferase failed to activate the pathway (negative data not shown). The difference in the BMP response seen between panels A and B in Figure 5 is a reflection of the size of the wells used in each experiment. We noticed that at 14 µM concentration, 4′-hydroxychalcone reduced the overall level of Smad protein which may be due to toxicity. Although the mechanism by which the two chalcones activate BMP signaling remains unclear, the observed high levels of P-Smad1, 5, Id1 and Id2 protein expression most likely results from inhibition of degradation rather than activation of receptor activity and might be explained by the modulation of their ubiquitination and turnover.

BMPs can also signal through Smad-independent mechanisms, including the MAPK pathway [39]. We assessed the activation of BMP signaling independently of P-Smad1, 5 by analyzing Extracellular Signal-Regulated protein Kinase (Erk) phosphorylation in C33A-2D2 cells treated or not with compounds at 5 and 10 µM. We detected an induction of phosphorylated Erk1/2 (P-Erk1/2) for isoliquiritigenin, 4′-hydroxychalcone and apigenin by immunoblotting (Figure S2A) that was quantified by Image J analysis (Figure S2B). The identification of apigenin as an activator of the MAPK pathway is in agreement with previously published results, since apigenin induces apoptosis in human rheumatoid arthritis fibroblast-like synoviocytes (RA-FLSs) [40], the main constituent of the aggressive front that can invade and destroy local articular structure in rheumatoid arthritis patients [41]. The effect of apigenin on RA-FLSs is mediated by a large increase of intracellular reactive oxygen species; which in turn causes activation of Erk1/2. Moreover, in RA-FLSs, apigenin induced phosphorylation of Erk1/2, without affecting p38 MAPK and JNK phosphorylation [40]. Apigenin and diosmetin that did not induce P-Smad1, 5 could act in a P-Smad-independent fashion. For example, prostacyclin analogs can induce Id1 without affecting Smad1, 5, 8 phosphorylation in a cAMP-dependent manner [42]. It is possible that a similar mechanism of action is responsible for the effects we observe with the two flavones identified in the screen.

### Osteoblastic differentiation of mouse myoblasts

**Figure 6:**
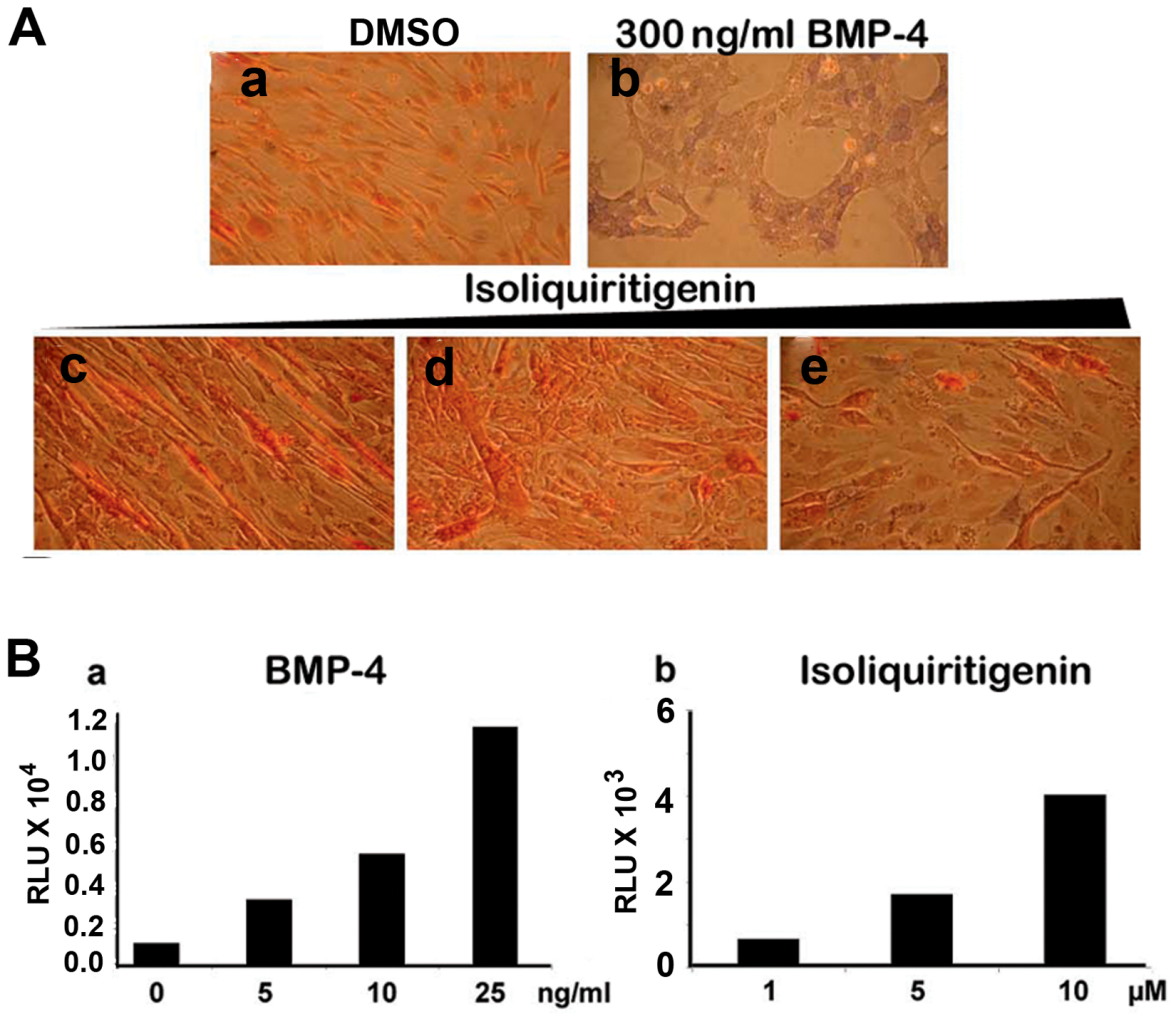
Alkaline phosphatase activity in C2C12 cells. (**A.**) Immunohistochemical staining with naphtol AS-BI alkaline and FBB-alkaline solution on C2C12 cells treated for 6 days with (**a**) DMSO; (**b**) 300 ng/ml BMP-4; (**c**) 1 µM; (**d**) 5 µM and (**e**) 20 µM isoliquiritigenin. (**B.**) ALP activity assay using an ALP luminescent substrate for quantification of data (Sensolyte^®^, AnaSpec). (**a**) BMP-4 and (**b**) Isoliquiritigenin.

C2C12 is a mouse myoblast cell line [43], [44] that rapidly differentiates into osteoblasts in response to BMP-4 [45]. One of the hallmarks of osteoblast induction is the expression of alkaline phosphatase (ALP) [46]. ALP is induced in primary osteoblasts by BMP-4 [47]. Although it is widely accepted that BMPs induce ALP in osteoblasts, both canonical (Smad-dependent) and non-canonical (Smad-independent) signaling have been shown to induce ALP in C2C12 cells [39], [48]. Isoliquiritigenin was the only flavonoid that partially differentiated C2C12 cells into osteoblasts compared to BMP-4 treated cells (Figure 6A) although it failed to activate Smad1, 5 phosphorylation or Id1 and Id2 expression in this cell line. Isoliquiritigenin induced an intermediate phenotype between those observed with DMSO and BMP-4 treatment, suggesting that it is less potent than BMP. This result was confirmed by increasing expression levels of ALP in a dose-dependent manner, above DMSO control, providing further functional evidence that this compound is a BMP signaling activator (Figure 6B). Quantification of ALP was performed by measuring luminescence from cleavage of a chemiluminescent substrate (Sensolyte ALP kit, Anaspec). The other chalcone, 4′-hydroxychalcone and the two flavonoid analogs luteolin and biochanin A diacetate did not induce ALP, as expected (negative data not shown).

### Activators of BMP signaling ventralize zebrafish embryos

**Figure 7:**
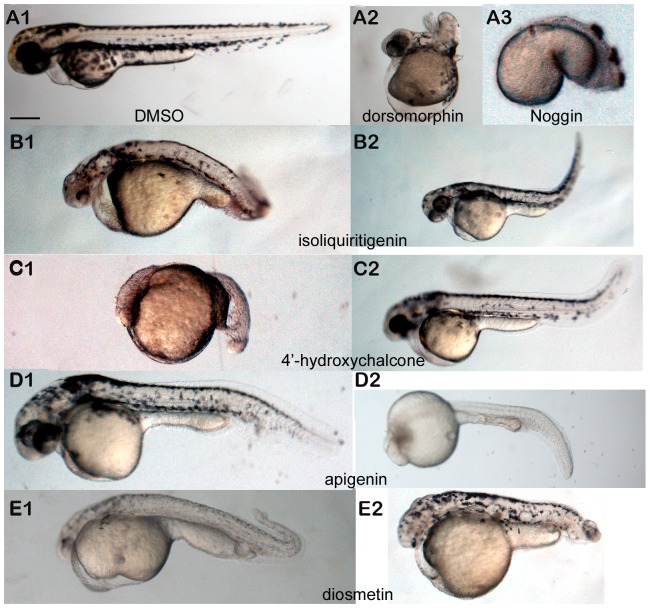
Effects of small molecules on zebrafish embryonic development. (A1.) Zebrafish embryos were treated from 2 to 50 hpf with DMSO as a negative control, (**A2.**) with 10 µM dorsomorphin to dorsalize the embryos, or (**A3.**) injected with a chordin morpholino to ventralize the embryos. (**B-E.**) treatment with 5 and 10 µM isoliquiritigenin (**B1,2**), 5 and 10 µM 4′-hydroxychalcone (**C1, 2**), 10 and 20 µM apigenin (**D1, 2**), and 5 and 10 µM diosmetin (**E1, 2**) ventralized the embryos. Embryos were imaged at 50 hpf. At least 15 animals were analyzed per concentration and per compound. Images represent the phenotype found in ≥33% of animals in the respective groups. Arrow indicates the loss of the ventral tail fin. Scale bar, 500 µm.

Zebrafish provide an ideal tool to functionally analyze BMP signaling. Small molecule inhibitors of BMP signaling dorsalize embryos [26], [49]. Whereas mutants and morphants for *bmp2b*, *bmp7*, and *smad5* dorsalize embryos [50], mutants and morphants for *chordin* ventralize embryos instead [29]. A ventralized phenotype is recognized by decreased to absent dorsal tissues (i.e. brain and eyes), and an abnormal accumulation of cells caudal to the urogenital pore visible at the 26-somite stage [22 hours post fertilization, (hpf)] [51]. In some cases, including the chordin-null mutant, multiple ventral fin folds are observed [52]. We tested whether the four small molecule flavonoids ventralized zebrafish embryos as would be predicted for BMP pathway agonists by treating wild-type embryos from 2 to 50 hpf. All four compounds induced ventralization defects (Figure 7, Table 1). They all induced a decrease in brain size, and treatment with 10 µM 4′-hydroxychalcone, 20 µM apigenin, 10 or 20 µM diosmetin (Figure 7, panel C1, D2 and E1 and E2) resulted in loss of eyes. Treatment with isoliquiritigenin resulted in an abnormal accumulation of cells caudal to the anus (Figure 7B2), and apigenin induced loss of the ventral tail fin (Figure 7D2). Increasing concentrations of compounds in each case caused a more severe phenotype (Figure 7B-E, 1 versus 2). Treatment with DMSO alone failed to affect embryonic development (Figure 7A1) whereas addition of dorsomorphin dorsalized the embryos (Figure 7A2), as previously reported [26]. Injection of chordin morpholino ventralized the embryos (Figure 7A3), as shown previously [29]. To determine if these effects were specific to all flavonoids, we tested compounds from the same chemical class and with similar structures that failed to induce luciferase in the primary assay, biochanin A, luteolin and biochanin A diacetate. They all failed to ventralize zebrafish embryos (Table 1, negative data not shown). These data suggest that ventralization of zebrafish embryos was induced by the two chalcones that induced BMP signaling but not by all chemicals from the class of the flavonoids [53], [54].

In summary, we have established a cell-based reporter assay that identified small molecules activators of BMP signaling. Our results suggest that high throughput screening of a large library of small molecules may identify novel activators of BMP signaling that could be useful in the treatment of medulloblastoma and also for other tumor types, including pediatric germ cell tumors. Because the currently available SHH antagonists lead to bone defects in young mice and to resistance in patients treated with GDC-0449 due to mutations in SMO [24], a combinatorial approach of SHH-antagonists with BMP agonists, possibly at lower doses to minimize side effects, might have therapeutic potential.

## Supporting Information

Figure S1 **Luciferase activity in response to BMP-4 in C3H10T1/2 cells.** C3H10T1/2 mouse embryonic mesenchymal cells were treated for 24 hours with a dilution series (1/3) of BMP-4. 24 hrs later, luciferase was measured as raw luminescent activity (RLU) using Steady-Lite Glo. The calculated EC_50_ was 8 ng/ml (95% confidence interval was 6–14 ng/ml). The dose-response curve for BMP-4 was not completely saturated at the highest concentration of 300 ng/ml BMP-4.
(DOC)

Figure S2 **Activation of MAPK signaling.** C33A-2D2 cells were treated with 5 µM of compound for 30 minutes, 4, 8 and 24 hours, and with 10 µM of compound for 24 hrs or for 8 hours with 10 ng/ml BMP-4 as positive control, or DMSO as negative control. Protein lysates were immunoblotted with antibodies to P-Erk1/2 and Erk1/2. Representative images are shown for each compound, quantification was performed using Image J analysis software, relative expression levels of P-Erk from 3 independent experiments are shown.
(DOC)
