# Supplementary material for: Identification of Small Molecule Activators of BMP Signaling
Source: PLoS One. 2013 Mar 19;8(3):e59045. doi: 10.1371/journal.pone.0059045 (PMC3602516; doi:10.1371/journal.pone.0059045)
Supplement: Figure S1 — Luciferase activity in response to BMP-4 in C3H10T1/2 cells. C3H10T1/2 mouse embryonic mesenchymal cells were treated for 24 hours with a dilution series (1/3) of BMP-4. 24 hrs later, luciferase was measured as raw luminescent activity (RLU) using Steady-Lite Glo. The calculated EC50 was 8 ng/ml (95% confidence interval was 6–14 ng/ml). The dose-response curve for BMP-4 was not completely saturated at the highest concentration of 300 ng/ml BMP-4. (DOC) [file pone.0059045.s001.doc]

**Supporting Information Figure S1**
