# Supplementary figures and images for: Identification of Small Molecule Activators of BMP Signaling
Source: PLoS One. 2013 Mar 19;8(3):e59045. doi: 10.1371/journal.pone.0059045 (PMC3602516; doi:10.1371/journal.pone.0059045)

**Supporting Information Figure S2**


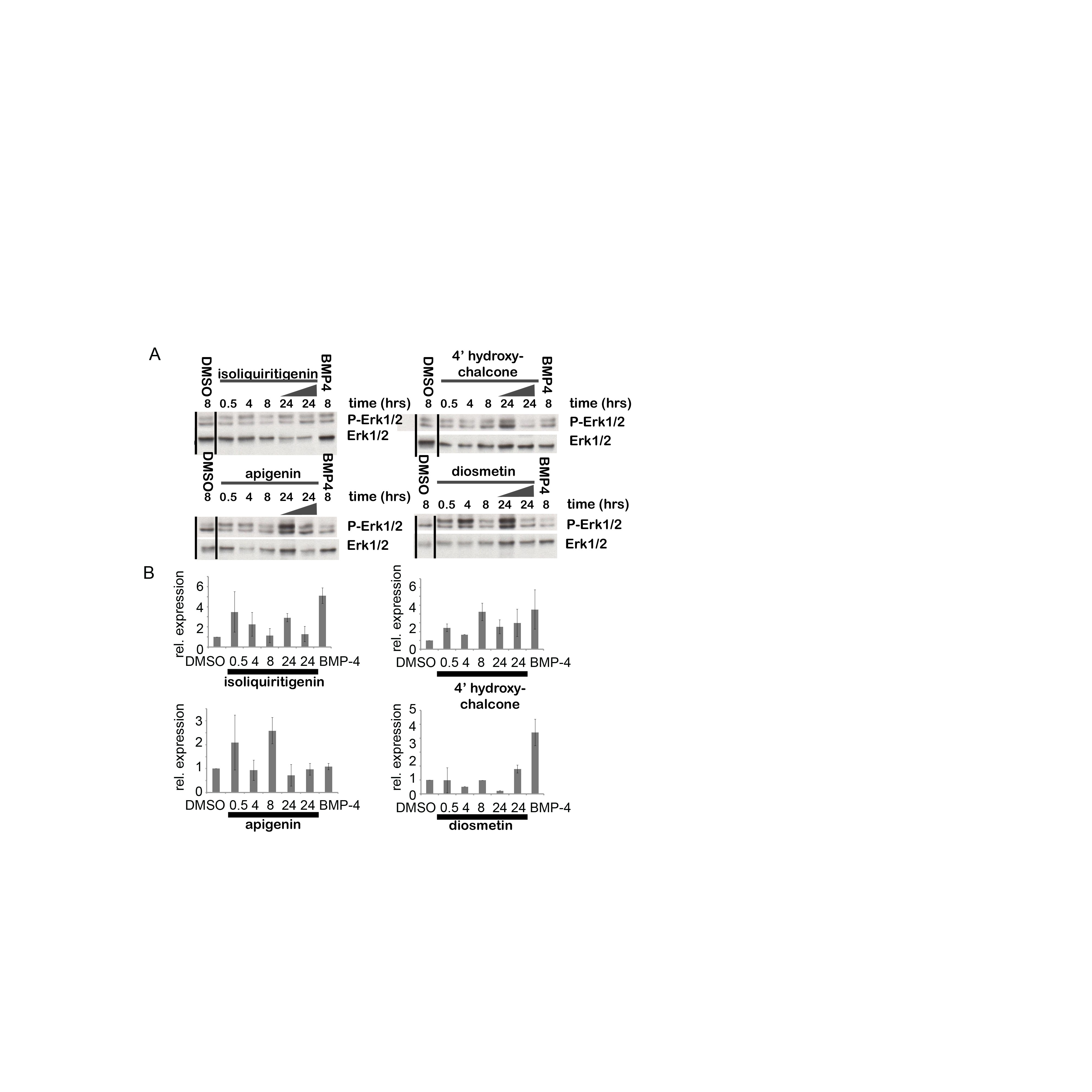

Supplement: Figure S2 — Activation of MAPK signaling. C33A-2D2 cells were treated with 5 µM of compound for 30 minutes, 4, 8 and 24 hours, and with 10 µM of compound for 24 hrs or for 8 hours with 10 ng/ml BMP-4 as positive control, or DMSO as negative control. Protein lysates were immunoblotted with antibodies to P-Erk1/2 and Erk1/2. Representative images are shown for each compound, quantification was performed using Image J analysis software, relative expression levels of P-Erk from 3 independent experiments are shown. (DOC) [file pone.0059045.s002.doc]
